# Computed tomography-guided biopsy of breast lesions: a viable option for selected cases

**DOI:** 10.1590/0100-3984.2019.0113

**Published:** 2020

**Authors:** Vinicius Cardona Felipe, Luciana Graziano, Paula Nicole Vieira Pinto Barbosa, Almir Galvão Vieira Bitencourt

**Affiliations:** 1 Imaging Department, A.C.Camargo Cancer Center, São Paulo, SP, Brazil.

## INTRODUCTION

The imaging methods of choice to guide percutaneous biopsies of suspected breast lesions are mammography, ultrasound, and magnetic resonance imaging (MRI)^(1)^. However, in some cases it may be difficult to perform a biopsy due to the characteristics of the lesion and its location. This occurs mainly in lesions identified only at MRI since biopsy guided by this method is still an expensive procedure with limited availability in Brazil^(2)^.

Computed tomography (CT)-guided percutaneous biopsy is a well-established technique for diagnosing lesions in different parts of the body^(3)^. Although few reports in the literature address the use of this technique for performing breast lesion biopsies, CT guidance is possible as long as the lesion is well characterized by this method. Despite the additional radiation dose, this procedure is faster and less expensive than MRI-guided biopsy^(4,5)^.

This study aimed to present the technique of CT-guided breast biopsy, which can be useful in selected cases when it is not possible or technically feasible to guide the biopsy by conventional methods.

## PROCEDURE

We describe two cases where a CT-guided large-core needle biopsy of breast lesions was performed. The procedures were performed jointly by medical radiologists who are specialized in breast imaging and interventional radiology. The procedures were indicated after identifying the lesion in previous CT examinations and in response to the difficulty in performing biopsy by other methods. After consenting to the procedure, patients were placed in a supine or oblique position on the CT table with their arms raised, using silicone cushions to adjust their position. Asepsis and local anesthesia were performed, followed by the introduction of a semi-automatic cutting needle, removal of four to six fragments for histological analysis, and placement of a metal clip. Sequential tomographic acquisitions were used to evaluate the progression and final positioning of the needle. No complications were observed.

**Figure 1:**
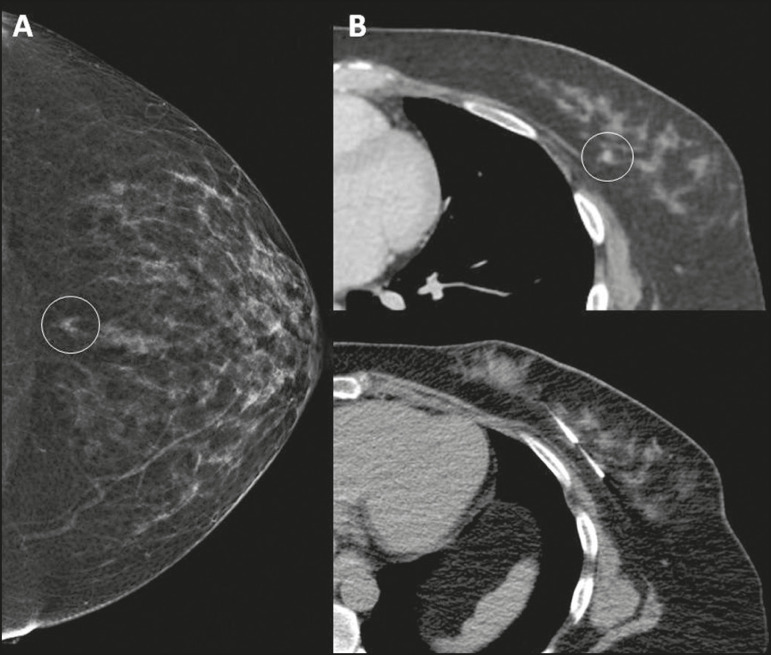
**A:** Mammography showing focal asymmetry in the posterior depth of the inner quadrants of the left breast (circle). **B:** CT showing a enhancing mass at the same topography of the mammographic finding (circle) and needle positioned to perform the biopsy.

In the first case, a 50-year-old patient showed a focal asymmetry in the posterior depth of the inner quadrants of the left breast on mammography, which was not characterized on ultrasound (Figure 1). A stereotactic-guided biopsy was attempted, but access was not possible due to the lesion location. A previous chest CT scan of the patient showed a enhancement mass in the same topography of the mammographic finding. A CT-guided biopsy was performed, and the histological result was invasive tubular carcinoma.

**Figure 2:**
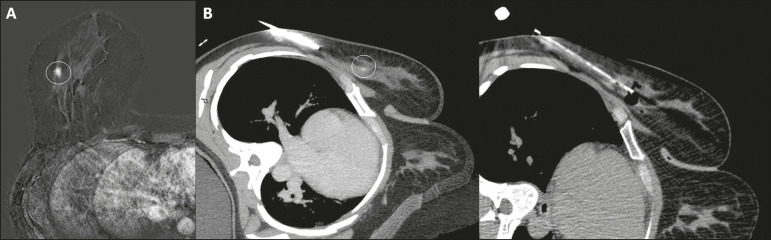
**A:** MRI showing a mass with irregular margins in the outer quadrants of the right breast (circle). **B:** CT showing the same mass in the right breast (circle) and needle positioned to perform the biopsy.

In the second case, a 39-year-old patient with cancer in the left breast underwent pretreatment MRI, which showed a new lesion in the right breast, not characterized by ultrasound and mammography (Figure 2). The patient had undergone a chest and abdomen CT scan for systemic staging, which showed the enhancing lesion in the right breast. Thus, a CT-guided procedure was performed. The histological result showed invasive ductal carcinoma.

## CONCLUSION

CT-guided large-core needle biopsy proved to be a safe and effective method for sampling breast lesions in selected cases where it is not possible to perform the procedure by conventional methods.
